# The impact of cortisol in steatotic and non‐steatotic liver surgery

**DOI:** 10.1111/jcmm.13156

**Published:** 2017-04-04

**Authors:** María Eugenia Cornide‐Petronio, Esther Bujaldon, Mariana Mendes‐Braz, Cindy G. Avalos de León, Mónica B. Jiménez‐Castro, Ana I. Álvarez‐Mercado, Jordi Gracia‐Sancho, Juan Rodés, Carmen Peralta

**Affiliations:** ^1^ Institut d'Investigacions Biomèdiques August Pi i Sunyer (IDIBAPS) Barcelona Spain; ^2^ Transplant Biomedicals S.L. Barcelona Spain; ^3^ Barcelona Hepatic Hemodynamic Laboratory IDIBAPS CIBEREHD Barcelona Spain; ^4^ Liver Unit Hospital Clínic Barcelona Spain; ^5^ Centro de Investigación Biomédica en Red de Enfermedades Hepáticas y Digestivas Barcelona Spain

**Keywords:** Partial hepatectomy, ischaemia–reperfusion, cortisol, acetylcholine, liver, steatosis

## Abstract

The intent of this study was to examine the effects of regulating cortisol levels on damage and regeneration in livers with and without steatosis subjected to partial hepatectomy under ischaemia–reperfusion. Ultimately, we found that lean animals undergoing liver resection displayed no changes in cortisol, whereas cortisol levels in plasma, liver and adipose tissue were elevated in obese animals undergoing such surgery. Such elevations were attributed to enzymatic upregulation, ensuring cortisol production, and downregulation of enzymes controlling cortisol clearance. In the absence of steatosis, exogenous cortisol administration boosted circulating cortisol, while inducing clearance of hepatic cortisol, thus maintaining low cortisol levels and preventing related hepatocellular harm. In the presence of steatosis, cortisol administration was marked by a substantial rise in intrahepatic availability, thereby exacerbating tissue damage and regenerative failure. The injurious effects of cortisol were linked to high hepatic acethylcholine levels. Upon administering an α7 nicotinic acethylcholine receptor antagonist, no changes in terms of tissue damage or regenerative lapse were apparent in steatotic livers. However, exposure to an M3 muscarinic acetylcholine receptor antagonist protected livers against damage, enhancing parenchymal regeneration and survival rate. These outcomes for the first time provide new mechanistic insight into surgically altered steatotic livers, underscoring the compelling therapeutic potential of cortisol–acetylcholine–M3 muscarinic receptors.

## Introduction

In clinical settings, partial hepatectomy (PH) under ischaemia–reperfusion (I/R) is a common strategy in use to limit bleeding during parenchymal dissection 1. More than 20% of patients destined for liver resection present with some degree of steatosis, a condition typically linked to obesity 1, 2, 3, and this figure is expected to rise in the near future. Hepatic steatosis is a major risk factor for surgery, associated with high rates of complications and postoperative mortality 1, 2, 4. By identifying mechanisms responsible for the failure of steatotic livers in this setting, discovery of new pharmacologic therapeutics may thus be advanced.

Cortisol is the main active hormone of the hypothalamic–pituitary–adrenal (HPA) axis, released by the adrenal gland in response to adenocorticotropic hormone (ACTH) 5, 6. Cortisol concentration in liver is regulated by the following enzymes: (*i*) 11 β‐hydroxysteroid dehydrogenase type 1 (11β‐HSD1), generating cortisol from inactive cortisone; (*ii*) 11β‐HSD2, converting cortisol to cortisone; and (*iii*) 5α‐ and 5β‐reductase enzymes, involved in cortisol clearance 5, 7. In pathologic states, adipose tissue may also display considerable endocrine activity, secreting a range of hormones including cortisol which may be taken up from the circulation by the liver 8, 9, 10. Disturbances of the HPA axis at any level or in hepatic cortisol metabolism and/or intrahepatic cortisol accumulation from circulation may be critical in the pathogenesis of various liver diseases and inflammatory disorders, including inflammatory bowel disease, colitis, atherosclerosis and metabolic syndrome 11, 12, 13.

Cortisol mediates anti‐inflammatory effects and programmed cell death in liver diseases such as cholestatic hepatitis, shock and sepsis 14, 15. It also stimulates hepatocellular proliferation in instances of PH without vascular occlusion 16. Hence, benefits may be anticipated if cortisol is given in surgical conditions requiring both liver regeneration and protection against I/R.

Several studies have suggested that in cultured human cells, the effects of cortisol rely upon the acethylcholine (ACh) signalling pathway 17, 18. ACh is the major neurotransmitter of vagus nerve and has proved beneficial in experimental models of sepsis, liver transplantation, haemorrhagic shock, myocardial ischaemia and pancreatitis 19, 20, 21. In addition, *in vitro* studies indicate that ACh stimulates an array of cell lines to proliferate 22, 23. However, it is not known whether such effects are exerted *via* binding to α7 nicotinic or M3 cellular receptors.

In this study, we examined hepatic cortisol levels, assessed changes in hepatic cortisol metabolic enzymes and determined the relevance of systemic adipose tissue as a major source of cortisol in the context of PH under I/R. We also evaluated the role of hepatic cortisol in rats with and without steatosis, subjected to PH under I/R, by investigating underlying ACh‐driven molecular effects.

## Materials and methods

### Experimental animals

The following groups of male Zucker rats were used: homozygous obese (Ob) animals with severe macro‐ and microvesicular hepatic fatty infiltration (400–450 g, 60–70% steatosis) or with mild‐to‐moderate hepatic fatty infiltration (250–300 g, 20–30% steatosis) and heterozygous lean (Ln) animals (350–400 g). All procedures were performed under isoflurane anaesthesia and were approved by the Laboratory Animal Care and Use Committee of the University of Barcelona. European Union regulations (Directive 86/609 EEC) for animal experiments were respected.

### Surgical procedure

Experimentation involved a rat model of PH (70%) under 60 min. of ischaemia (standard duration in liver surgery), as described elsewhere 24, 25. Briefly, once isoflurane anaesthesia was achieved and the left hepatic lobe resected, a microvascular clamp was applied across portal triad (supplying median lobe) for 60 min. Bowel congestion (due to clamping) was avoided by maintaining portal flow through right and caudate lobes. At the end of the ischaemic period, both right and caudate lobes were resected, and the median lobe was reperfused by releasing the clamp 24, 25.

### Experimental design

#### Protocol 1: Cortisol metabolism/signalling and effects of cortisol in non‐steatotic and steatotic livers (60–70% steatosis)

Procedural subsets were as follows: (*i*) dissection of hepatic hilar vessels (sham); (*ii*) PH (70%); (*iii*) PH (70%) + I/R (60‐min. ischaemic period), standard throughout; (*iv*) PH + I/R after initial lipectomy of retroperitoneal and epididymal white adipose tissue (PH + I/R + LPT) 25; (*v*) PH + I/R + cortisol [5 mg/kg, intraperitoneal (IP)] 10 min. preoperatively 16; (*vi*) PH + I/R + ACh [500 μg/kg, intravenous (IV)] 10 min. preoperatively 19; (*vii*) PH + I/R + α7 nAChR antagonist (5 mg/kg, IP) 30 min. preoperatively 19; (*viii*) PH + I/R + M3 mAChR antagonist (1 mg/kg, IP), 30 min. preoperatively 26; and (*ix*) PH + I/R + cortisol (5 mg/kg, IP) 10 min. preoperatively 16 and M3 mAChR antagonist (1 mg/kg, IP) 30 min. preoperatively (PH + I/R + cortisol + M3 mAChR antagonist) 26.

#### Protocol 2: Effects of cortisol and ACh in steatotic livers (20–30% steatosis)

Procedural subsets (1–4) duplicated subsets 1, 3, 5 and 6, respectively, of Protocol 1.

In all instances, plasma, liver (median lobe) and white adipose tissue were sampled 16 and 24 hrs postoperatively, freezing specimens on dry ice for biochemical, molecular biology and oil red staining or immersing in formalin for histologic studies. Hepatic injury (assessed by transaminase levels and damage score) and regeneration [per cent Ki 67‐positive hepatocytes as well as hepatocyte growth factor (HGF) and transforming growth factor (TGF‐β) expression] were gauged at these time‐points. To address survival times, select Ln and Ob rats subjected to Protocol 1 (subsets 3–9) were monitored for 14 days 25. Doses and treatment times were based on prior studies 16, 19, 25, 26 and on preliminary experimentation done by our group.

### Biochemical determinations

Alanine aminotransferase (ALT), aspartate aminotransferase (AST), glutamate dehydrogenase (GLDH), HGF and total and active TGF‐β levels were quantified as described elsewhere 25, 27. HGF is a potent mitogen, and active TGF‐β is considered the principal inhibitor of hepatocellular proliferation 25. Cortisol and ACTH levels were measured by enzyme‐linked immunosorbent assay (Bionova Científica, Madrid, Spain). Commercial kits (BioVision Inc, Mountain View, CA, USA) were used for ACh determinations. Myeloperoxidase (MPO), an index of neutrophilic influx; malondialdehyde (MDA); nitrotyrosines (reflecting oxidative stress); and hepatic oedema were measured as described elsewhere 28, 29.

### Western blotting

Hepatic tissues were processed to obtain cytosolic fractions 25. Western blotting was conducted as described elsewhere 15, 19, using antibodies directed against the following proteins: 11β‐HSD1, 11β‐HSD2, 5αR1, 5αR2, 5βR, α7 nAChR, M3 AChR and phosphoinositide‐3‐kinase (PI3K; Cell Signaling Technology, Danvers, MA, USA); total and phosphorylated Akt (T‐Akt and p‐Akt, respectively; Santa Cruz Biotechnology, Dallas, TX, USA); and β‐actin (Sigma‐Aldrich, St Louis, MO, USA). Immunoreactive protein bands were visualized through chemiluminescence, and densitometric quantification relied on standard software (Quantity One; Bio‐Rad Laboratories, Hercules, CA, USA).

### Histology, Oil Red O staining and immunohistochemistry

To gauge severity of hepatic injury, haematoxylin and eosin (H&E)‐stained sections were graded as follows: (0) no or minimal injury; (1) mild injury (*i.e*. cytoplasmic vacuolation and focal nuclear pyknosis); (2) moderate or severe injury with extensive nuclear pyknosis, cytoplasmic hypereosinophilia and loss of intercellular bridges; (3) severe necrosis, marked by hepatic cord disintegration, haemorrhage and neutrophilic infiltrates; and (4) very severe necrosis, showing the latter manifestations to extreme degree 25. Hepatic steatosis was assessed *via* Oil Red O staining of frozen sections, with percentages rendered from standard image analysis 25. Hepatic samples analysed for regeneration were immunostained using rabbit monoclonal antibody to Ki 67 (clone SP6; Abcam, Cambridge, UK), diaminobenzidine (for colorization) and haematoxylin counterstain 25. At least 30 high‐power fields were counted per slide.

### Statistics

Statistical significance of differing variables was determined *via* nonparametric Kruskal–Wallis test. Mann–Whitney *U*‐test was applied to groups showing significant differences, and adjusted *P*‐values were calculated using false discovery rate (FDR) method (*P*.adj<0.05 considered significant). Survival estimates, obtained by Kaplan–Meier method, were then compared using log‐rank test, with statistical significance set at *P* < 0.05.

## Results

### Cortisol levels in livers with and without steatosis (60–70%) 16 hrs postoperatively

**Figure 1:**
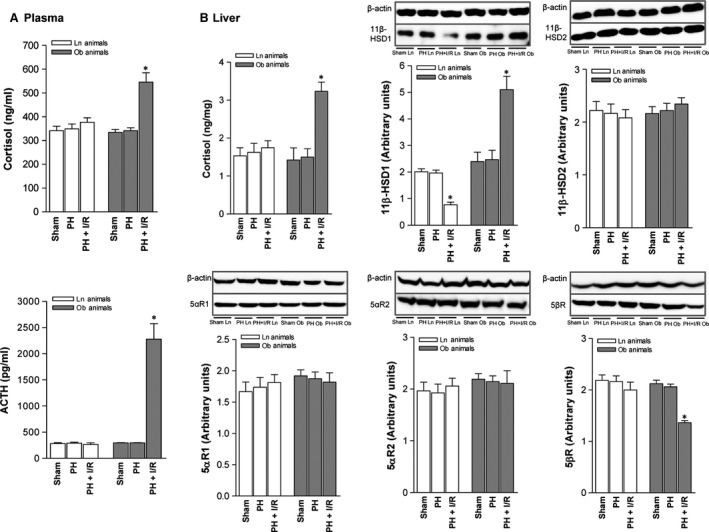
Cortisol levels in non‐steatotic and steatotic livers (60–70% steatosis) 16 hrs postoperatively: (**A**) cortisol and ACTH levels in plasma and (**B**) assay of cortisol and enzymes regulating cortisol metabolism in liver (representative Western blots, top; densitometric analysis, bottom). Data from six lean and six obese animals, per group, *P < 0.05 *versus* sham.

In Ln and Ob rats, cortisol and ACTH levels and enzymatic expression involved in cortisol metabolism were similar for PH and sham procedural subsets (Fig. 1A and B). Plasma cortisol and ACTH levels (Fig. 1A) and hepatic cortisol levels (Fig. 1B) in the PH + I/R subset of Ln (non‐steatotic) rats were similar to those of sham procedural counterparts. Although enzymes regulating cortisol inactivation or clearance (*i.e*. 11β‐HSD2, 5αR and 5βR) also were unchanged postoperatively, 11β‐HSD1 (generating cortisol) declined (Fig. 1B). However, plasma cortisol and ACTH levels in the PH + I/R subset of steatotic rats were higher than those found in the sham subset (Fig. 1A), as were hepatic cortisol levels (Fig. 1B). Under these conditions, enzymes regulating cortisol production (11β‐HSD1 specifically) were overexpressed, and those involved in cortisol clearance (such as 5βR) were downregulated (Fig. 1B).

**Figure 2:**
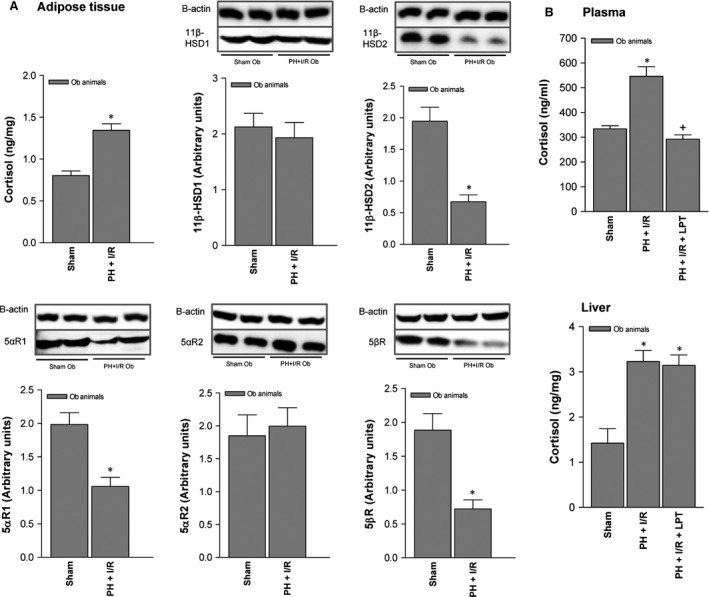
Cortisol levels in adipose tissue of Ob Zucker rats (60–70% steatosis) 16 hrs postoperatively: (**A**) assay of cortisol and enzymes regulating cortisol metabolism in adipose tissue (representative Western blots, top; densitometric analysis, bottom) and (**B**) cortisol levels in plasma and liver after lipectomy. Data from six obese animals per group. *P < 0.05 *versus* sham; +*P* < 0.05 *versus* PH + I/R.

Given that adipose tissue may be a real source of various hormones, including cortisol in certain pathologic states 8, 9, 10, we examined whether high circulating and hepatic cortisol levels found in Ob rats subjected to PH under I/R may have originated from systemic adipose tissue. Cortisol levels in adipose tissue of the Ob PH + I/R subset were higher than corresponding levels in the sham subset (Fig. 2A), but enzymes engaged in cortisol inactivation or clearance, including 11β‐HSD2, 5αR1 and 5βR, were decreased (Fig. 2A). Surgical removal of adipose tissue in Ob rats (PH + I/R + LPT) reduced cortisol levels in plasma (but not in liver), relative to the PH + I/R subset (Fig. 2B).

### Impact of cortisol on hepatic damage and regenerative failure, with and without steatosis (60–70%) 16 and 24 hrs postoperatively

**Figure 3:**
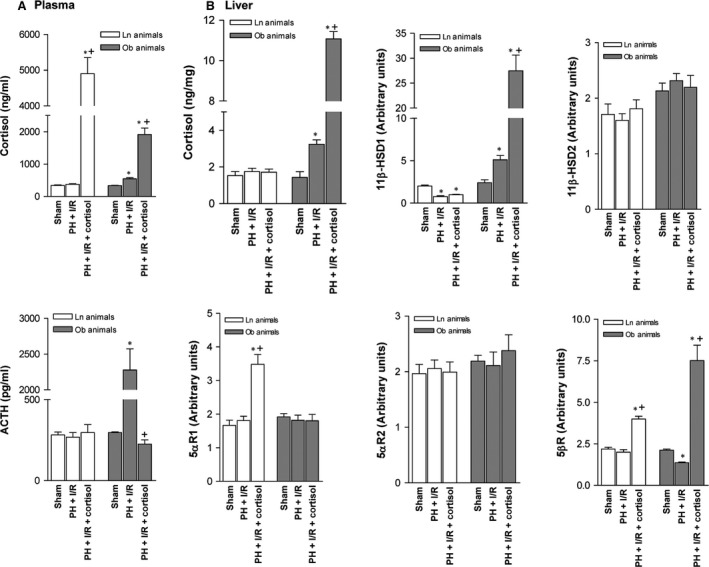
Effects of cortisol administration on cortisol metabolism in liver (non‐steatotic and steatotic) 16 hrs postoperatively: (**A**) cortisol and ACTH levels in plasma and (**B**) assay of cortisol and enzymes regulating cortisol metabolism in liver, non‐steatotic and steatotic. Data from six lean and six obese animals, per group; *P < 0.05 *versus* sham; *P* < 0.05 *versus* PH + I/R.

Although cortisol given prior to PH under I/R increased plasma cortisol levels in Ln rats, ACTH levels were unchanged (Fig. 3A), and relative to PH under I/R alone, cortisol levels in non‐steatotic livers were unaffected (Fig. 3B). Enzymes involved in cortisol clearance (including 5αR1 and 5βR) were elevated in this setting (Fig. 3B). Adding cortisol to PH under I/R in Ob rats increased plasma cortisol levels but reduced ACTH levels, relative to PH under I/R alone (Fig. 3A). Cortisol levels in steatotic livers were boosted by cortisol administration (PH + I/R + cortisol; Fig. 3B). Under these conditions, expression of 11β‐HSD1 (promoting cortisol generation) increased, as did expression of enzymes such as 5βR (involved in cortisol clearance).

**Figure 4:**
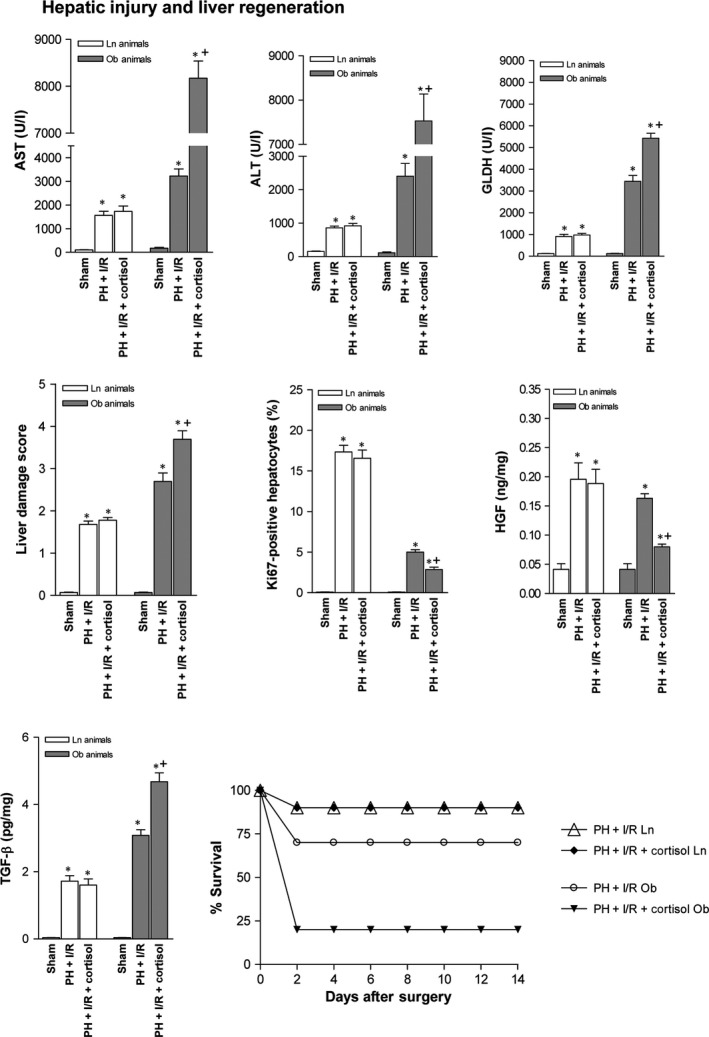
Effects of cortisol administration on tissue damage and regeneration in liver (non‐steatotic and steatotic) 16 hrs postoperatively: hepatic injury (plasma AST, ALT and GLDH levels; damage score) and hepatic regeneration (per cent Ki‐67 positivity; HGF and TGF‐β levels). Data from six lean and six obese animals, per group. Survival of 10 lean and 10 obese animals, per group was monitored (postoperative day 14). *P < 0.05 *versus* sham; +*P* < 0.05 *versus* PH + I/R.

**Figure 5:**
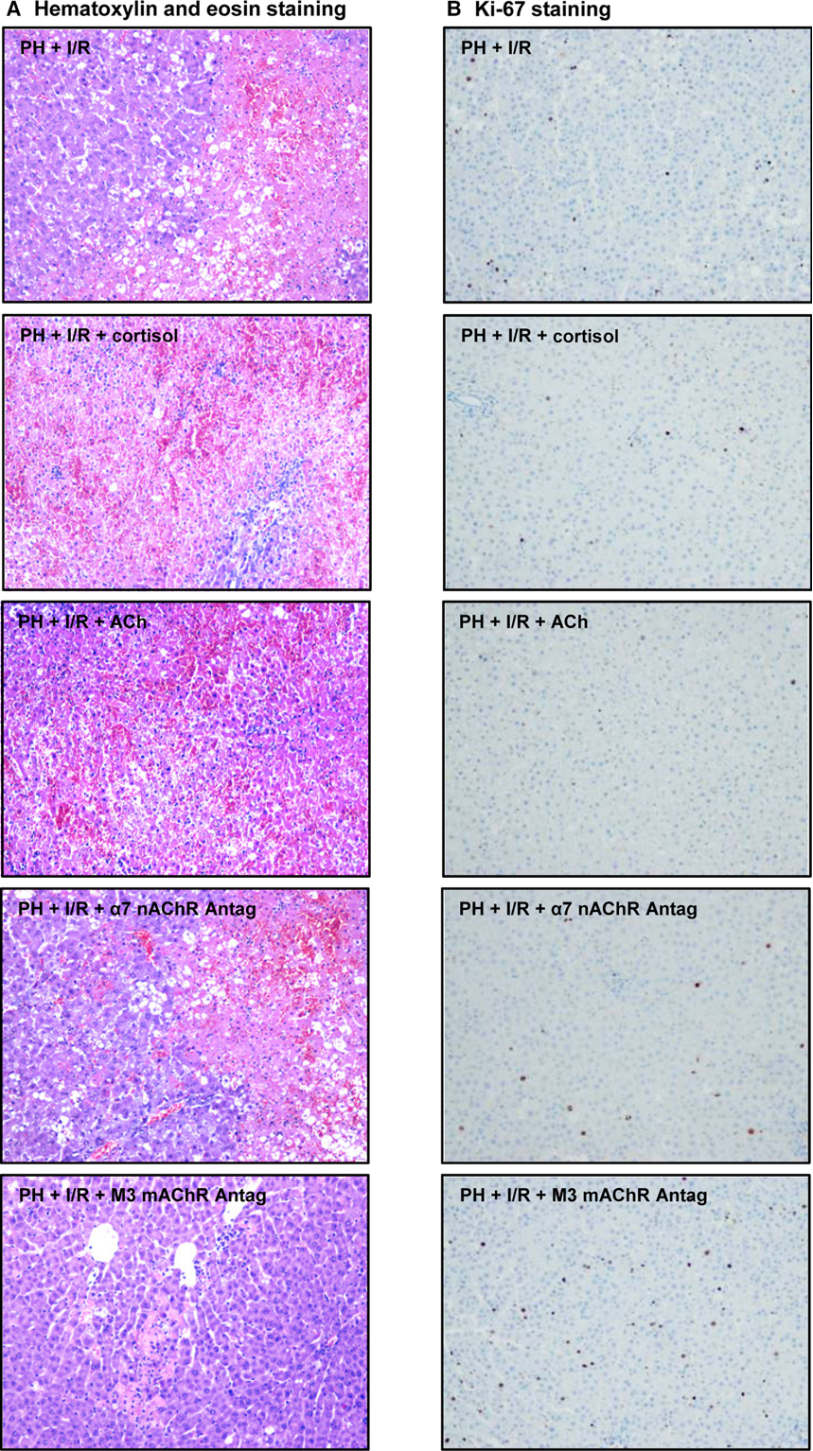
Histologic features and Ki‐67 positivity in steatotic livers (60–70%) 16 hrs postoperatively: (**A**) representative photomicrographs of extensive coagulative necrosis seen in most procedural subsets but very limited in subset PH + I/R + M3 mAChR antagonist (H&E stain, 10×) and (**B**) representative photomicrographs of Ki‐67 immunohistochemical positivity, strongest in hepatocytes of PH + I/R + M3 AChR antagonist procedural subset (10 × ).

Cortisol administration prior to PH under I/R in Ln rats did not alter either liver damage or regeneration (Fig. 4). In fact, transaminase concentrations, GLDH levels and damage scores were similar to those for PH under I/R alone, as were Ki‐67 positivity assessments, levels of HGF and active TGF‐β and animal survival rates. All groups had similar total hepatic TGF‐β levels (data not shown). However, in steatotic livers, cortisol injection (PH + I/R) increased transaminase and GLDH levels and raised damage scores, extending necrotic areas ordinarily seen after PH under I/R (Figs 4 and 5A). Extensive, confluent areas of coagulative necrosis developed postoperatively, with neutrophilic infiltration (Fig. 5A), and there were significantly fewer Ki 67‐positive hepatocytes by comparison (Figs 4 and 5B). This reduction in proliferative cells was also associated with low levels of HGF and high levels of active TGF‐β (Fig. 4). Subsequent analysis showed a 70% survival rate (3 of 10 dead) for the PH + I/R subset of Ob rats at 14 days (Fig. 4), compared with 20% survival (8 of 10 dead) at 14 days for the PH + I/R + cortisol subset of Ob rats.

Given that markers of hepatic damage peak relatively early (16 hrs) after reperfusion, followed by signs of hepatocyte proliferation (24 hrs), we then confirmed that hepatic injury and regeneration due to cortisol were similar for both time frames (Figs 4 and 6 ‐ 16 and 24 hrs, respectively).

**Figure 6:**
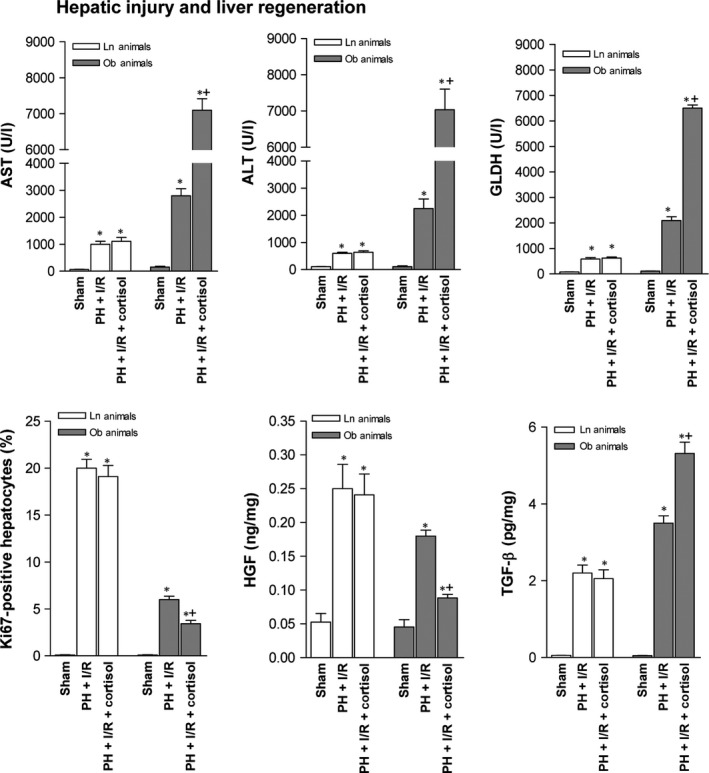
Effects of cortisol administration on tissue damage and regeneration in non‐steatotic and steatotic livers (60–70%) 24 hrs postoperatively: hepatic injury (plasma AST, ALT and GLDH levels) and hepatic regeneration (per cent Ki‐67 positivity; HGF and TGF‐β levels). Data from six lean and six obese animals, per group; *P < 0.05 *versus* sham; +*P* < 0.05 *versus* PH + I/R.

### Effects of cortisol‐induced ACh accumulation on tissue damage and regeneration in steatotic livers (60–70%) 16 hrs postoperatively

**Figure 7:**
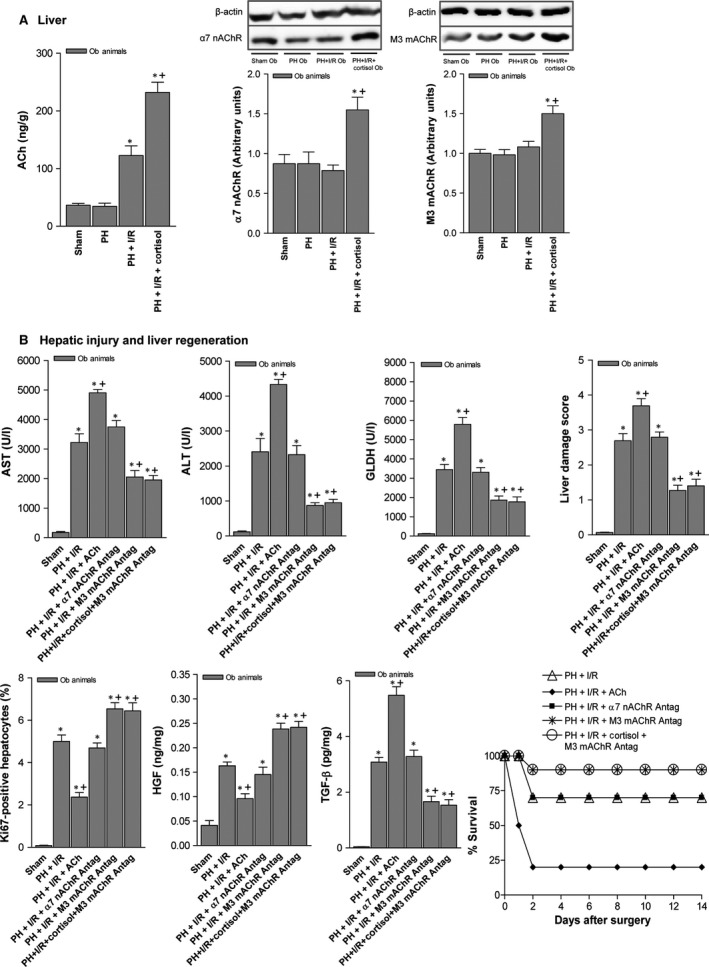
Effects of cortisol‐induced ACh accumulation on tissue damage and regeneration in steatotic livers (60–70%) 16 hrs postoperatively: (**A**) ACh, α7 nicotinic and M3 muscarinic acetylcholine receptor levels in liver (representative Western blots, top; densitometric analysis, bottom) and (**B**) hepatic injury (plasma AST, ALT and GLDH levels; damage score) and regeneration (per cent Ki‐67 positivity; HGF and TGF‐β levels). Data from six obese animals per group; survival of 10 obese animals per group (**B**), postoperative day 14); *P < 0.05 *versus* sham; +*P* < 0.05 *versus* PH + I/R.

Acethylcholine levels in non‐steatotic livers of the PH + I/R subset were similar to those of the sham subset (40.73 ± 15.33 *versus* 39.44 ± 9.55 ng/g tissue, *P* > 0.05, data not shown in graphs). As confirmed in Figure 7A, PH under I/R (not PH alone) increased ACh levels in steatotic livers, compared with sham surgery. An attempt was made to discern whether ACh levels might account for the harmful hepatocellular effects of cortisol in steatotic livers subjected to PH under I/R. Cortisol administration produced a relative rise in ACh levels of steatotic livers, thereby increasing hepatic expression of both α7 nicotinic and M3 muscarinic AChR (Fig. 7A). However, ACh administration prior to PH under I/R (*versus* PH under I/R alone) also increased hepatic injury, with fewer Ki 67‐positive hepatocytes, low levels of HGF and high active TGF‐β levels (Fig. 7B). Mortality in Ob rats (8 of 10 dead, 20% survival rate at 14 days) was also increased by adding ACh to PH under I/R (Fig. 7B). Still, an α7 nAChR antagonist (PH + I/R + α7 nAChR antagonist) did nothing to mitigate manifestations of hepatic damage or regeneration in this setting, indicating a lesser role for α7 nAChR. In contrast, administering a M3 mAChR antagonist alone or in combination with cortisol (PH + I/R + M3 mAChR antagonist or PH + I/R + cortisol + M3 mAChR antagonist) did confer protection in steatotic livers, reducing transaminase and GLDH levels, limiting the frequency and extent of necrotic areas and lowering damage scores, relative to corresponding markers for PH under I/R (Figs 5 and 7B). Likewise, Ki 67‐positive hepatocytes showed a proportionate increase, associated with high HGF and low TGF‐β levels. Consequently, the survival rate in Ob rats rose to 90% (1 of 10 dead) at 14 days (Fig. 7B).

**Figure 8:**
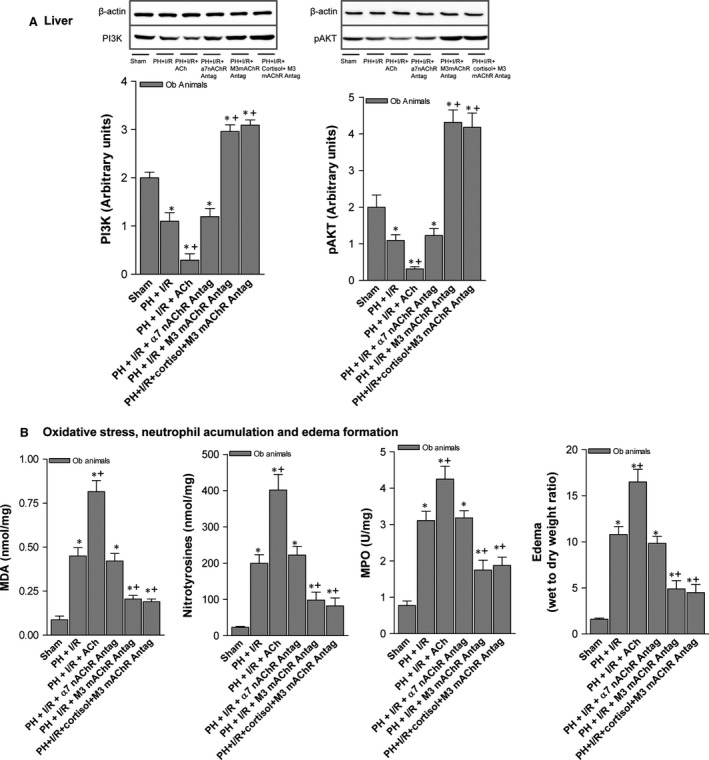
ACh mechanisms of action in steatotic livers (60–70%) 16 hrs postoperatively: (**A**) hepatic PI3K/pAkt expression (representative Western blots, top; densitometric analysis, bottom) and (**B**) MDA, nitrotyrosine and MPO levels and hepatic oedema. Data from six obese animals, six per group. *P < 0.05 *versus* sham; +*P* < 0.05 *versus* PH + I/R.

Based on published insights into the mechanisms of action for ACh 30, we chose to evaluate whether ACh affects the PI3K/Akt pathway in resected steatotic livers. Hepatic levels of proteins PI3K and pAkt (the primary target of PI3K‐initiated signalling) were lowered by adding ACh to PH under I/R (Fig. 8A), inducing relative increases in MDA, nitrotyrosine and MPO levels and fostering hepatic oedema (Fig. 8B). Addition of an M3 mAChR antagonist instead, with or without cortisol administration (PH + I/R + M3 mAChR antagonist or PH + I/R + cortisol + M3 mAChR antagonist), was associated with PI3K/Akt overexpression, thus protecting steatotic livers from oxidative stress, neutrophil influx and oedema.

### Effects of cortisol and ACh on tissue damage and regeneration in steatotic livers (20–30%) 16 hrs postoperatively

**Figure 9:**
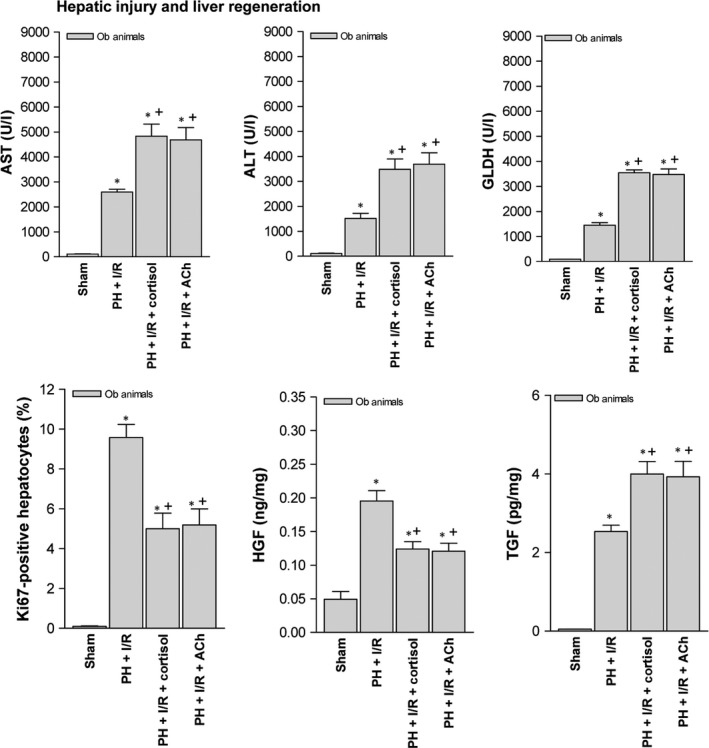
Effects of cortisol and ACh on tissue damage and regeneration in steatotic livers (20–30%) 16 hrs postoperatively: hepatic injury (plasma AST, ALT and GLDH levels) and hepatic regeneration (per cent Ki‐67 positivity; HGF and TGF‐β levels). Data from six obese animals, six per group; *P < 0.05 *versus* sham; +*P* < 0.05 *versus* PH + I/R.

As in highly steatotic livers (60–70% steatosis), added cortisol or ACh increased biochemical markers of hepatic damage, relative to PH under I/R alone (Fig. 9). Significantly fewer Ki 67‐positive hepatocytes resulted from cortisol or ACh injection as well, with low levels of HGF and high levels of active TGF‐β.

## Discussion

The findings reported here are the first to suggest that, in the context of PH under I/R, cortisol administration has no virtually impact on non‐steatotic livers, whereas steatotic livers are actually harmed by added cortisol. This is in sharp contrast with the known therapeutic benefits of cortisol in cholestatic hepatitis, shock and sepsis 14, 15. However, such pathologic states differ fundamentally 14, 15 from surgical intervention, so this is not entirely surprising, and as with other treatments 4, 31, 32, both surgical strategy (PH alone or with vascular occlusion) and baseline liver status (steatotic *versus* non‐steatotic) may ultimately dictate the hepatocellular effects of cortisol. Studies of non‐steatotic livers subjected to PH without I/R indicate that cortisol dosage <6.25 mg/kg stimulates hepatocytic proliferation, whereas higher cortisol dosing impairs the regenerative process 16, 33. Our experimental model of PH was adapted accordingly, using a therapeutic cortisol dosage of 5 mg/kg. Of note, we found that low‐dose cortisol (<5 mg/kg) did not raise either circulating or hepatic cortisol levels in the setting of PH + I/R surgical procedures (data not shown). On the other hand, the benefits of cortisol in event of PH 16 were reversed by implementing vascular occlusion (PH + I/R). Furthermore, the changes in cortisol metabolism/signalling that we observed in steatotic livers subjected to PH under I/R were not primarily attributable to PH. The nature of hepatic and surgical conditions, through specific mechanisms of hepatic I/R injury and regeneration, resulted in differing outcomes for the various therapeutic strategies evaluated.

From our perspective, cortisol‐induced ACh accumulation may partly explain the harmful effects of administering cortisol prior to resecting steatotic liver. ACh (*via* M3 muscarinic receptors) reduced PI3K/Akt expression and exacerbated the inflammatory response, thus worsening tissue damage and encouraging regenerative failure. Indeed, the role of ACh does differ in various pathologic states, proving advantageous in haemorrhagic shock, myocardial ischaemia and pancreatitis 19, 20, 21, but promoting inflammation and damage in chronic obstructive pulmonary disease 34. Our results for PH under I/R are nonetheless unlike than those detailed in previous reports of liver transplants from brain‐dead donors, whereby steatotic grafts were protected by ACh during prolonged (6‐hrs) cold ischaemia 35. This is expected because the systemic alterations induced by brain death (absent during PH under I/R), the extent and type of ischaemia (*i.e*. cold or warm) and the presence of regeneration all lead to differences in the mechanisms of hepatic I/R injury and in the effects of the therapeutic strategies evaluated.

Regarding the changes in cortisol levels and cortisol metabolism/signalling in the surgery of hepatic resections, we report by the first time that, unlike Ln rats, whose cortisol levels were unchanged by liver surgery, hepatic and circulating cortisol levels increased in Ob rats subjected to PH under I/R. Hence, a net increase in secretory activity of HPA axis was displayed by Ob rats only. The low levels of cortisol observed postoperatively in non‐steatotic livers were associated with reduced 11β‐HSD1, and upon cortisol administration, clearance of hepatic cortisol predominated in such livers, driven by increased reductase expression. Therefore, a compensatory mechanism is apparently operant, preventing intrahepatic cortisol overload and its deleterious hepatocellular effects. Postoperative cortisol accumulation in remnant steatotic livers seemed to occur independently, unrelated to peripheral adipose tissue sources, perhaps due in part to altered enzymatic regulation of cortisol metabolism. As in some chronic inflammatory conditions, such as non‐alcoholic steatohepatitis, inflammatory bowel disease and colitis 12, 36, overexpression of 11β‐HSD1 and reductase downregulation have been confirmed in steatotic livers subjected to surgery. Administration of cortisol prompts overexpression of 5βR (ostensibly to eliminate excess hepatic cortisol), which may be offset by high levels of 11β‐HSD1. Hence, accumulated cortisol reaped its harmful effects in steatotic livers.

In the event of deficient 11β‐HSD1 secretion (impairing cortisol replenishment), the tendency for plasma cortisol to decline is pre‐empted through enhanced ACTH‐dependent cortisol production 5. Conversely, impaired peripheral inactivation of cortisol (*e.g*. 11β‐HSD2 deficiency) is balanced *via* HPA axis, reducing cortisol production sufficiently to maintain normal plasma levels 5. It then appears that enzymatic fluctuations important in determining intracellular cortisol levels of steatotic livers do not lessen the importance of regulating circulating cortisol concentrations by HPA axis. The cortisol given to our Ob rats (prior to PH under I/R) triggered a negative feedback loop through HPA axis, lowering circulating ACTH levels (and ACTH‐dependent cortisol secretion by default) to counteract a surge in circulating cortisol. However, hepatic cortisol levels were not similarly modulated, with adverse parenchymal consequences. In non‐steatotic livers, endogenous cortisol metabolism seemed to prevail, modulating intracellular cortisol locally, whereas the role played by HPA axis (regulating circulating cortisol levels) was minor. Cortisol administration did not alter circulating ACTH levels. It is quite feasible that endogenous hepatic enzymes regulate cortisol metabolism independently, keeping cortisol levels low and limiting any harmful effects.

Investigations aimed at identifying prognostic factors in liver surgery are both necessary and relevant. Date presented here indicate that not only is there no direct association between circulating levels and extent of hepatic damage or regeneration failure, but also hepatic enzymes governing cortisol metabolism may vary, without corresponding changes in plasma cortisol levels. For this reason, it is unlikely that cortisol will become an important prognostic factor in liver surgery.

Further studies beyond present scope are needed to determine why circulating cortisol levels were lower in Ob (*versus* Ln) rats after cortisol administration. We feel that differential effects of cortisol on the HPA axis may explain the disparities of this animal model, rooted in baseline liver status. However, potential contributions by adipose tissue or liver in Ob rats (through either increased uptake of cortisol from circulation or by reduced circulatory release) after cortisol administration cannot be discounted. Ultimately, hepatic cortisol levels were higher in Ob (*versus* Ln) rats following cortisol injection.

**Figure 10:**
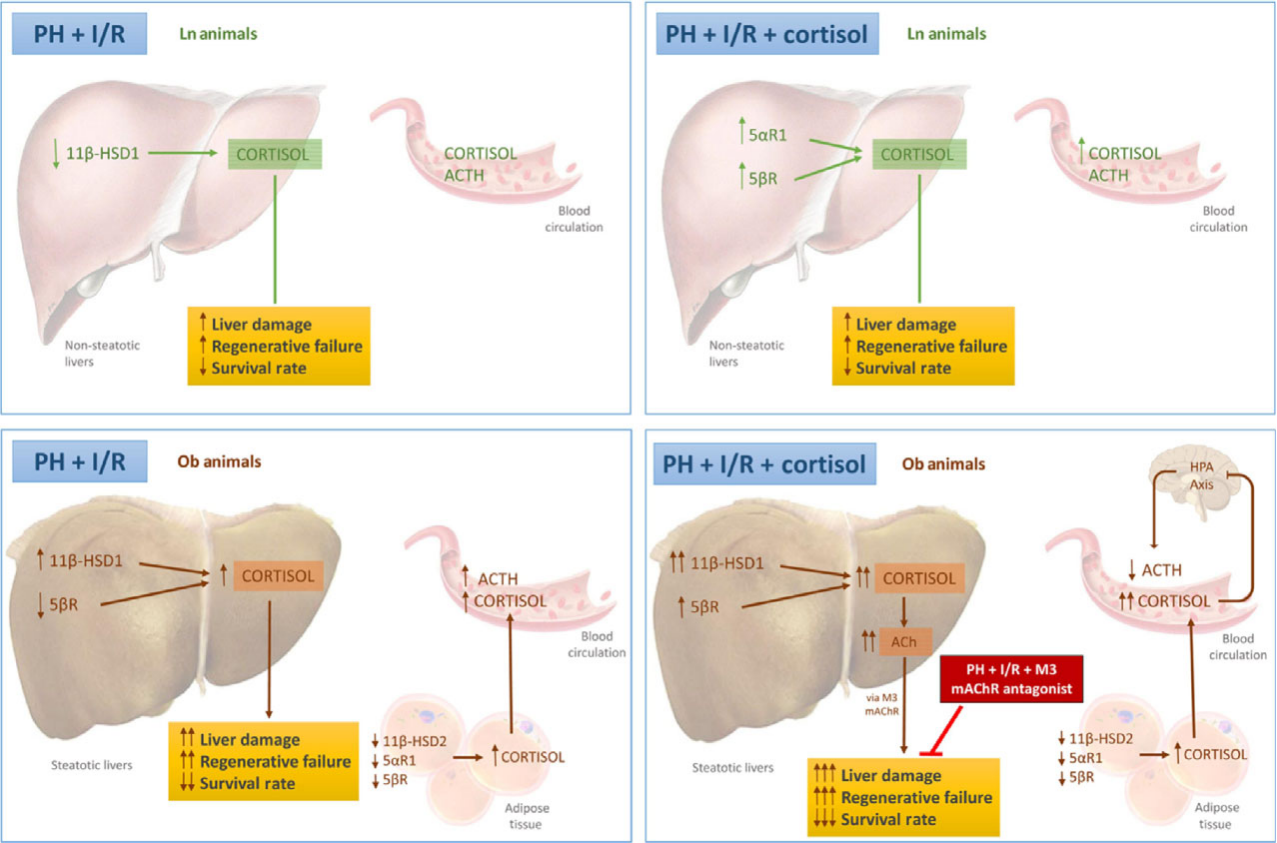
Schematic of various procedural and pharmacologic interventions conducted (PH + I/R; PH + I/R + cortisol; and PH + I/R + M3 mAChR antagonism), depicting outcomes and proposed signalling pathways.

Here, we report that in instances of PH under I/R, HPA axis fundamentals, hepatic cortisol levels, enzymatic shifts in hepatic cortisol metabolism and the contributory potential of adipose tissue (as a cortisol source) are all dependent on baseline liver status. Non‐steatotic livers seem capable of limiting cortisol excess, preventing adverse manifestations that otherwise ensue. In steatotic livers, the opposite is true. Cortisol levels are poorly controlled, culminating in worse tissue damage and regenerative failure postoperatively (Fig. 10). Strategies to block cortisol activity are probably worthwhile to protect steatotic livers in this setting, given that cortisol in hepatic remnants may favour accumulation of ACh. It is our contention that in the presence of hepatic steatosis, blockade of M3 mAChR may reduce the incidence of postoperative complications following PH under vascular occlusion.

## Author contributions

MECP and EB contributed to acquisition of experimental data and data analysis/interpretation. MMB, CGAL, MBJC and AIAM all contributed to acquisition of experimental data. JGS and JR contributed to data analysis/interpretation and critical revision of this article. CP contributed to the study concept/design, acquisition of experimental data, data analysis/interpretation and drafting and critical revision of this article. All authors have approved the final manuscript.

## Conflict of interest

The authors declare that they have no conflict of interest.
